# Analysis of the Relationship between Early Clinical Factors and Glasgow Outcome Scale in Patients With Traumatic Brain Injury

**DOI:** 10.1002/brb3.71703

**Published:** 2026-08-29

**Authors:** Wanghan Luo, Xiaoyun Cheng, Weiguo Ying

**Affiliations:** ^1^ Department of Emergency and Critical Care Yiwu Fuyuan Private Hospital Yiwu City China; ^2^ Department of Intensive Care Unit Yiwu Fuyuan Private Hospital Yiwu City China

**Keywords:** early clinical factors, Glasgow outcome scale, prognosis, traumatic brain injury

## Abstract

**Objective:**

This study aimed to evaluate the association between early clinical factors and the Glasgow outcome scale (GOS) in patients with traumatic brain injury (TBI).

**Methods:**

We conducted a retrospective analysis of 98 TBI patients who underwent emergency surgery between January 2021 and January 2024. Based on GOS scores at 6 months post‐surgery, patients were classified into a favorable outcome group (GOS ≥ 4, defined as moderate disability or good recovery, *n* = 58) and an unfavorable outcome group (GOS < 4, i.e., death, persistent vegetative state, or severe disability, *n* = 40). Baseline and early clinical parameters were compared between groups. Statistically significant variables from univariate analysis were entered into a multivariate logistic regression model to identify independent prognostic factors.

**Results:**

Significant intergroup differences were observed in age, time from injury to surgery, bleeding site, midline shift, Glasgow coma scale (GCS) score at admission, blood glucose level, and D‐dimer level (all *p* < 0.05). Multivariate analysis confirmed that age, time from injury to surgery, GCS score, blood glucose, and D‐dimer level were independent predictors of GOS (all *p* < 0.05).

**Conclusion:**

Early clinical factors, including age, time to surgery, GCS score, blood glucose, and D‐dimer level, independently influence GOS in TBI patients. Time from injury to surgery emerged as a potentially modifiable factor in this cohort, suggesting that minimizing delays may improve outcomes.

## Graphical abstract

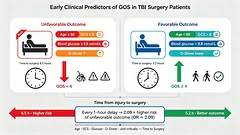

## 1 Introduction

Traumatic brain injury (TBI) involves mechanical damage to brain structures due to external forces, triggering inflammatory responses, apoptosis, and oxidative stress that impair central nervous system function (Maas et al. 2022). Globally, TBI affects an estimated 69 million individuals each year, representing a major cause of death and long‐term disability, with particularly high burdens in low‐ and middle‐income settings (Dewan et al. 2019). Although emergency surgery is a cornerstone of TBI management, a significant number of patients experience poor outcomes. Early identification of high‐risk individuals is crucial for optimizing treatment strategies (Gravesteijn et al. 2020). Previous research has emphasized serological and imaging markers, often neglecting routine clinical indicators that may affect emergency care. The influence of the time interval from injury to surgery remains controversial, while factors such as age, injury type, and severity also require further investigation (Gravesteijn et al. 2020). This study retrospectively applied univariate and multivariate logistic regression to analyze the relationship between early clinical factors and GOS, aiming to inform clinical practice.

## 2 Materials and Methods

### 2.1 Study Population

Inclusion criteria were (1) confirmed TBI diagnosis; (2) admission GCS score ≤ 12; (3) age ≥ 18 years; and (4) emergency craniotomy for hematoma evacuation. Exclusion criteria included (1) cerebrospinal fluid leakage; (2) severe polytrauma; (3) major organ dysfunction; (4) prior cranial surgery or cerebral infarction; (5) known coagulation disorders, defined as documented history of inherited bleeding disorders, current use of anticoagulant or antiplatelet medications, or laboratory evidence of severe coagulopathy (e.g., platelet count < 100 × 10^9^/L or INR > 1.5 when available). However, INR, PT, and APTT were not routinely measured in all patients and thus were not included as study variables. In the absence of complete coagulation panels, the exclusion of coagulopathy relied primarily on clinical history and platelet count; (6) intraoperative death; and (7) incomplete 6‐month follow‐up. Medical histories of hypertension and diabetes mellitus were not consistently documented across records and therefore could not be included in the analysis. A total of 98 patients (69 male and 29 female), aged 22–74 years, were enrolled from January 2021 to January 2024. Injury mechanisms included traffic accidents (61 cases), falls from height (11 cases), and ground‐level falls (26 cases). The study was approved by the institutional ethics committee.

### 2.2 Grouping

The Glasgow outcome scale is a five‐point scale: 1 = death, 2 = persistent vegetative state, 3 = severe disability (conscious but dependent on daily support), 4 = moderate disability (independent but with some disability), and 5 = good recovery (resumption of normal life with possible minor deficits). A favorable outcome was defined as GOS ≥ 4 (moderate disability or good recovery) because this threshold captures patients who regain functional independence, a clinically meaningful endpoint widely adopted in TBI prognostic studies (Hawryluk et al. 2019). Patients were stratified into a favorable outcome group (GOS ≥ 4, *n* = 58) and an unfavorable outcome group (GOS < 4, *n* = 40) based on 6‐month postoperative GOS assessment (Hawryluk et al. 2019).

### 2.3 Data Collection

Data extracted from medical records comprised demographics, injury cause, admission blood pressure, hematoma volume, platelet count, hemoglobin, D‐dimer level, time from injury to surgery, GCS score, basal cistern status, midline shift, bleeding location, and admission glucose level. Variables included in well‐validated prognostic models, such as pupillary reactivity, the motor component of the GCS, and detailed radiographic classifications (e.g., Rotterdam CT score), were not available in this dataset. Biochemical and hematological analyses were performed using a Roche Cobas C450 autoanalyzer and a Mindray BS2000 hematology analyzer, respectively. GOS was assessed at 6‐month follow‐up.

### 2.4 Statistical Analysis

Data were analyzed using SPSS 25.0. Continuous variables were expressed as mean ± standard deviation and compared via *t*‐tests; categorical variables were presented as counts (percentages) and compared using chi‐square tests. Variables with *p* < 0.05 in univariate analysis were included in a multivariate logistic regression model to identify independent prognostic factors. Multicollinearity was assessed using the variance inflation factor (VIF), with VIF > 10 indicating significant collinearity. Statistical significance was set at *p* < 0.05. Categorical variables were entered into the logistic model with the following reference groups: age < 60 years, GCS > 8, and blood glucose < 8.8 mmol/L. Continuous variables (time to surgery and D‐dimer) were analyzed per unit increase.

## 3 Results

### 3.1 Comparison of Early Clinical Factors between Groups

Significant differences were found between the favorable and unfavorable outcome groups in age, time from injury to surgery, bleeding site, midline shift, GCS score, blood glucose, and D‐dimer level (all *p* < 0.05). Details are provided in Table 1.

**TABLE 1:** Comparison of early clinical factors between groups.

Factor	Favorable (*n* = 58)	Unfavorable (*n* = 40)	*p*‐value
Age ≥ 60 years, *n* (%)	17 (29.3%)	26 (65.0%)	0.02
Male, *n* (%)	40 (69.0%)	29 (72.5%)	0.75
BMI (kg/m^2^)	23.40 ± 3.25	23.29 ± 3.18	0.49
Injury cause, *n* (%)			0.27
Traffic accident	36 (62.1%)	25 (62.5%)	
Fall from height	6 (10.3%)	5 (12.5%)	
Ground‐level fall	16 (27.6%)	10 (25.0%)	
GCS ≤ 8 at admission, *n* (%)	27 (46.6%)	33 (82.5%)	0.01
SBP (mmHg)	146.28 ± 17.97	147.84 ± 18.52	0.72
DBP (mmHg)	87.54 ± 10.41	87.91 ± 10.76	0.48
Basal cistern compression, *n* (%)	42 (72.4%)	30 (75.0%)	0.73
Hematoma volume (mL)	31.19 ± 7.57	31.37 ± 7.70	0.26
Midline shift > 0.5 cm, *n* (%)	27 (46.6%)	29 (72.5%)	0.04
Bleeding site, *n* (%)			0.03
Epidural	22 (37.9%)	14 (35.0%)	
Subdural	30 (51.7%)	16 (40.0%)	
Multiple	6 (10.3%)	10 (25.0%)	
Blood glucose ≥ 8.8 mmol/L, *n* (%)	23 (39.7%)	30 (75.0%)	0.01
Platelet count (×10^9^/L)	198.85 ± 29.24	205.14 ± 31.30	0.42
Hemoglobin (g/L)	132.68 ± 15.60	134.09 ± 16.26	0.60
D‐dimer (ng/mL	9103.86 ± 2750.81	19825.10 ± 4316.83	0.03
Time to surgery (h)	5.21 ± 0.77	6.49 ± 1.15	0.01

### 3.2 Multivariate Logistic Regression Analysis of GOS

Multivariate analysis identified age, time from injury to surgery, GCS score, blood glucose, and D‐dimer level as independent factors significantly associated with 6‐month GOS (all *p* < 0.01). Multicollinearity was not a concern, with variance inflation factor (VIF) values ranging from 1.12 to 1.58 across all included variables. The Hosmer–Lemeshow test indicated good model fit (*χ* ^2^ = 6.38, *p* = 0.61), and the model showed satisfactory discriminative ability with an area under the receiver operating characteristic curve (AUC) of 0.84 (95% CI: 0.76–0.92). Results are shown in Table 2. Bleeding site and midline shift, although significant in univariate analysis, were not retained in the final multivariate model after adjustment for other covariates (forward stepwise selection, removal criterion *p* > 0.10).

**TABLE 2:** Multivariate logistic regression analysis of GOS.

Factor	*B*	SE	Wald *χ* ^2^	OR	95% CI	*p*‐value
Age (≥ 60 vs. <60 years)	0.81	0.31	6.83	2.25	1.22–4.10	0.009
Time to surgery (per 1‐h increase)	0.74	0.23	10.35	2.09	1.33–3.28	0.001
GCS at admission (≤ 8 vs. > 8)	1.04	0.25	17.31	2.83	1.72–4.65	< 0.001
Blood glucose (≥ 8.8 vs. < 8.8 mmol/L)	0.94	0.17	30.57	2.56	1.83–3.58	< 0.001
D‐dimer level (per 1000 ng/mL increase)	0.33	0.12	7.56	1.39	1.10–1.76	0.006

*Note*: Variables that were significant in univariate analysis (bleeding site and midline shift) were entered into the initial model but did not retain statistical significance after adjustment for other covariates (*p* > 0.05) and were therefore excluded by the forward stepwise selection procedure (entry criterion *p* < 0.05, removal criterion *p* > 0.10).

## 4 Discussion

Although the prognostic factors identified in this study—age, admission GCS, hyperglycemia, and D‐dimer elevation—are already well‐recognized outcome predictors in TBI, this analysis re‐emphasizes their relevance in a surgical cohort and, more importantly, highlights that a shorter time from injury to surgery is independently associated with improved 6‐month outcomes. The core predictors observed here align with those in established prognostic models such as IMPACT and CRASH, which integrate age, GCS (including motor score), pupillary reactivity, and CT characteristics (Steyerberg et al. 2008; Perel et al. 2008). By confirming these associations in a Chinese emergency surgical population, our data provide contextual validation. Notably, time from injury to surgery is not part of the IMPACT or CRASH models, and its independent effect in our cohort suggests it may serve as an additional modifiable prognostic factor that warrants consideration in acute management protocols.

Advanced age is a well‐established risk factor for poor TBI outcomes, with older patients exhibiting increased vulnerability to white matter injury, higher perioperative mortality, and elevated complication rates (Steyerberg et al. 2008; Gardner et al. 2018). Our results align with these findings, emphasizing the need for age‐adjusted risk assessment and management in emergency settings.

Time from injury to surgery emerged as a critical modifiable factor. Prolonged intervals are associated with elevated intracranial pressure, delayed neural recovery, and increased mortality (Silva et al. 2025). Early surgical intervention, when feasible, may significantly improve outcomes, highlighting the importance of efficient triage and resource allocation. In the present study, time to surgery was analyzed as a continuous variable; a clinically actionable threshold (e.g., ≤4 h) was not derived, and we did not examine whether the benefit of earlier evacuation differs by hematoma type or patient subgroup. Notably, the relationship between surgical timing and outcome remains complex—some studies have even reported that ultra‐early surgery (< 4 h) is associated with worse outcomes, likely reflecting confounding by injury severity, where the most critically ill patients undergo the most expeditious surgery (Yrysov et al. 2023). Future investigations with larger, stratified cohorts are needed to define optimal surgical windows and identify populations that gain the greatest benefit from timely intervention.

A lower GCS score at admission consistently correlates with worse neurological outcomes (Carney et al. 2017). According to the NICE head injury guidelines, GCS scores of 9–12 define moderate head injury, and scores of ≤8 define severe head injury (National Institute for Health and Care Excellence [NICE] 2023). In our cohort, 82.5% of patients in the unfavorable outcome group had admission GCS ≤ 8, confirming the established severity–outcome association. Our data reinforce the value of GCS as a key indicator for stratifying injury severity and predicting long‐term prognosis.

Elevated blood glucose and D‐dimer levels at admission were also independent predictors of unfavorable outcomes. Hyperglycemia exacerbates cerebral acidosis and inflammatory responses, impairing neuronal repair (Hermanides et al. 2018). Similarly, elevated D‐dimer levels reflect hyperfibrinolysis and coagulation activation, contributing to secondary brain injury and hemorrhage progression (Jin et al. 2023; Tian et al. 2010). Monitoring these parameters may aid in early identification of high‐risk patients and guide timely interventions.

This study has several limitations beyond its retrospective design and sample size. First, the data lacked several variables established as critical in contemporary prognostic models—most notably pupillary reactivity, the motor component of the GCS, detailed CT scoring systems, and complete coagulation profiles, including INR—which restricts direct comparability with frameworks like IMPACT and CRASH. Furthermore, according to the recently published Chinese expert consensus on the diagnosis and treatment of coagulation disorders in the acute phase of TBI (2024), standard coagulation tests including INR, PT, APTT, and platelet count are strongly recommended as routine assessments; the diagnosis of coagulation dysfunction requires at least one abnormal finding among these indicators (Yuan et al. 2024). The absence of INR, PT, and APTT data in our retrospective cohort therefore constitutes a major limitation that precludes a complete assessment of coagulation status and limits direct comparison with contemporary diagnostic standards. Second, surgical details (e.g., specific operative approach, surgeon experience, intraoperative blood loss, and operation duration) were not analyzed, and comorbidities such as hypertension and diabetes could not be adjusted for, potentially introducing confounding. Third, the small cohort relative to the number of screened variables raises concern for model overfitting. However, the VIF values (all < 2) indicated no significant multicollinearity, and the Hosmer–Lemeshow test suggested adequate model calibration. Nevertheless, the odds ratios should still be interpreted cautiously, and external validation is needed. Finally, the single‐center enrollment limits generalizability.

## 5 Conclusion

Early clinical factors, including age, time to surgery, GCS score, blood glucose, and D‐dimer level, independently influence GOS in TBI patients. Time from injury to surgery emerged as a potentially modifiable factor in our single‐center cohort, suggesting that minimizing delays may improve outcomes. However, given the limited and sometimes conflicting evidence regarding optimal surgical windows, these findings should be considered hypothesis‐generating and require validation in larger, prospective, multi‐center studies before being adopted as a general recommendation. This study functions primarily as a single‐center confirmation of established prognostic factors and highlights the value of surgical timing in real‐world emergency care. Multi‐center, prospective studies that incorporate pupillary reactivity, standardized coagulopathy panels (including INR, PT, and APTT as recommended by the 2024 Chinese consensus), detailed surgical data, and larger samples are required to validate these findings and build more robust, generalizable prognostic tools.

## Author Contributions

**Weiguo Ying**: investigation, validation, formal analysis, writing – review and editing, supervision, writing – original draft. **Wanghan Luo**: writing – original draft, writing – review and editing, project administration, conceptualization, resources, formal analysis. **Xiaoyun Cheng**: methodology, writing – review and editing, project administration, formal analysis, data curation, conceptualization.

## Ethics Statement

This study was approved by the Ethics Committee of the Yiwu Fuyuan Private Hospital (Approval No.: 2020ER012‐2). Due to the retrospective nature of the study and the use of anonymized data, the requirement for informed patient consent was waived by the Ethics Committee. All methods were carried out in accordance with Declaration of Helsinki.

## Funding

The authors have nothing to report.

## Declaration of Generative AI and AI‐Assisted Technologies

The authors confirm that no generative artificial intelligence (AI) tools were used in the creation of this manuscript. All analyses, writing, and revisions were performed by the authors.

## Conflicts of Interest

The authors declare no conflicts of interest.

## Data Availability

The datasets used and analyzed during the current study are available from the corresponding author upon reasonable request.
